# Ursodeoxycholic Acid Halts Pathological Neovascularization in a Mouse Model of Oxygen-Induced Retinopathy

**DOI:** 10.3390/jcm9061921

**Published:** 2020-06-19

**Authors:** Menaka C. Thounaojam, Ravirajsinh N. Jadeja, Shubhra Rajpurohit, Diana R. Gutsaeva, Brian K. Stansfield, Pamela M. Martin, Manuela Bartoli

**Affiliations:** 1 Department of Ophthalmology, Medical College of Georgia, Augusta University, Augusta, GA 30912, USA; shurajpurohit@augusta.edu (S.R.); dgutsaeva@augusta.edu (D.R.G.); mbartoli@augusta.edu (M.B.); 2 Department of Biochemistry and Molecular Biology, Augusta University, Augusta, GA 30912, USA; rjadeja@augusta.edu (R.N.J.); pmmartin@augusta.edu (P.M.M.); 3 Department of Pediatrics and Neonatal-Perinatal Medicine, Medical College of Georgia, Augusta University, Augusta, GA 30912, USA; bstansfield@augusta.edu; 4 Vascular Biology Center, Augusta University, Augusta, GA 30912, USA

**Keywords:** retinal neovascularization, bile acids, UDCA, retinopathy of prematurity

## Abstract

Retinopathy of prematurity (ROP) is the leading cause of blindness in infants. We have investigated the efficacy of the secondary bile acid ursodeoxycholic acid (UDCA) and its taurine and glycine conjugated derivatives tauroursodeoxycholic acid (TUDCA) and glycoursodeoxycholic acid (GUDCA) in preventing retinal neovascularization (RNV) in an experimental model of ROP. Seven-day-old mice pups (P7) were subjected to oxygen-induced retinopathy (OIR) and were treated with bile acids for various durations. Analysis of retinal vascular growth and distribution revealed that UDCA treatment (50 mg/kg, P7–P17) of OIR mice decreased the extension of neovascular and avascular areas, whereas treatments with TUDCA and GUDCA showed no changes. UDCA also prevented reactive gliosis, preserved ganglion cell survival, and ameliorated OIR-induced blood retinal barrier dysfunction. These effects were associated with decreased levels of oxidative stress markers, inflammatory cytokines, and normalization of the VEGF–STAT3 signaling axis. Furthermore, in vitro tube formation and permeability assays confirmed UDCA inhibitory activity toward VEGF-induced pro-angiogenic and pro-permeability effects on human retinal microvascular endothelial cells. Collectively, our results suggest that UDCA could represent a new effective therapy for ROP.

## 1. Introduction

Retinopathy of prematurity (ROP) is an ocular pathology affecting premature babies and is the leading cause of preventable blindness in children [1,2,3]. Complications of ROP include myopia, early development of cataracts, iris neovascularization, glaucoma, rhegmatogenous, and exudative retinal detachments [4,5,6,7]. Current therapies for ROP include laser photocoagulation, cryotherapy, and vitrectomy [8,9,10]. Intravitreal injections of anti-vascular endothelial growth factor (VEGF) are also used to halt retinal neovascularization (RNV) in the most advanced stages of ROP [8,9,10]. All of these therapeutic approaches involve surgical procedures with harmful side effects including significant loss of visual field, glaucoma, and others [8,9,10,11]. Despite encouraging pre-clinical studies, dietary supplementation with omega-3 polyunsaturated fatty acid (PUFA) in premature babies has been shown to partially prevent the progression of ROP only to the most severe stages [12]. Therefore, therapies for this sight-threatening disease are still needed and, ideally, should halt abnormal vascularization without affecting normal vasculogenesis of the postnatal developing retina.

Bile acids (BA) are amphipathic sterols that are primarily synthesized in the liver from cholesterol and released in the intestinal lumen upon food intake. BA play an important role in the elimination of cholesterol, micellination of dietary lipids to aid their transport, stimulation of bile flow, biliary phospholipid secretion, and finally, in negative feedback regulation of cholesterol synthesis [13]. However, emerging evidence suggests that aside from their conventional biological function, BA are also important signaling molecules [13]. In humans, the principle primary BA are cholic acid and chenodeoxycholic acid, and the main secondary bile acids are deoxycholic acid and lithocholic acid. Ursodeoxycholic acid (UDCA) and tauroursodeoxycholic acid (TUDCA), which are abundant in Asiatic and American black bears, are found only in trace amount in humans [14]. However, they have been extensively used as pharmacological agents and have shown to possess high biological activity in humans and exert a variety of protective effects [15,16,17]. Of interest, in the last decade, studies on the usefulness of these BA in the treatment of ocular diseases have gained great interest in the scientific community. UDCA and TUDCA are reported to be beneficial against photoreceptor degeneration, ganglion cell death, cataract, diabetic retinopathy, etc. [14,18]. Despite this evidence, the efficacy of BA for the treatment of ROP has not been previously investigated. In the present study, we aimed at investigating the efficacy and mode of action of the BA UDCA and its conjugated derivatives TUDCA and GUDCA (glycoursodeoxycholic acid) in an experimental model of ROP.

## 2. Experimental Section

### 2.1. Animal Model of Oxygen-Induced Retinopathy (OIR)

All the animal procedures were performed per the statement of the Association for Research in Vision and Ophthalmology (ARVO) and following animal protocols approved by The Augusta University Institutional Animal Care and Use Committee (Approval no: 2009-0181). C57BL/6J mice were purchased from Jackson Laboratories (Bar Harbor, ME) at 2 months of age and were maintained and bred in Augusta University animal facility (Augusta, GA, USA), with a light cycle of 12 h and fed ad libitum.

Oxygen-induced retinopathy (OIR), the experimental model of ROP, was applied in C57BL/6J mice according to the protocol described by Smith et al. [19] and previously used in our laboratory [20,21,22,23]. On postnatal day 7 (P7), newborn mice were placed, along with their dam, into a custom-built chamber in which the partial pressure of oxygen was maintained at 75% for 5 days to induce vaso-obliteration. At P12, the mice were brought to room air (21% oxygen) and kept until P17 to induce retinal neovascularization. To evaluate the effect on normal retinal development, a set of mice were kept at room air from days P0–17 (control age-matched mice).

### 2.2. Bile Acids Treatment

UDCA, GUDCA, or TUDCA (Sigma-Aldrich, St. Louis, MO, USA) were administered by intraperitoneal injection (i.p) at a dose of 50 mg/kg/day from P7–17 or P7–12 (UDCA only) or P12–17 (UDCA only). All the OIR treatment groups were exposed to 75% oxygen from P7–P12 and room air from P12–P17. Two groups of mice without BA treatments were either exposed to room air only (control) or served as an untreated OIR control and injected with vehicle (2.5% sodium bicarbonate; pH 7.4). At the end of the treatment, mice were sacrificed to collect eyes for further evaluation.

### 2.3. Central Vaso-Obliteration and Neovascularization

To assess retinal vessel growth and distribution, mice were euthanized at P17, and eyes were enucleated and fixed with 4% paraformaldehyde for 1 h. The whole retinal flat mounts were dissected and stained with biotinylated isolectin GS-IB4 from *Griffonia simplicifolia* (0.2 mg/mL; Invitrogen, Carlsbad, CA, USA) and Texas red–conjugated avidin D overnight at 4 °C [20,22]. The retinal flat-mounts were imaged with a Zeiss Axioplan 2 fluorescence microscope (Carl Zeiss, Thornwood, NY, USA) equipped with Axio Vision 4.6.3.0 software. Electronic images were processed using image-editing software (Adobe Photoshop; Adobe Systems, Inc., Mountain View, CA, USA) to create whole retina montages. The area of neovascularization was assessed by a semi-automated quantification method (SWIFT_NV) installed on ImageJ software (National Institute of Health, MD, USA) as developed and described by Stalh et al. [24]. Using SWIFT_NV macros, blinded user-delineated vascular tufts that were subsequently quantified in a semi-automated way based on fluorescence intensity thresholds were determined manually by the user. The percentage of the neovascular area over the total retinal area was calculated. The avascular areas were also measured and reported as percentage of the total retina area. Briefly, using a polygon selection, the total area and area with no vessel were marked in ImageJ. Later, the individual images were analyzed using the measure function embedded in ImageJ software to obtain total and avascular areas. For each experimental condition, quantitative data originated from six different retinas obtained from six different mice were analyzed in blind. After statistical analysis, the averaged data were plotted on the same graph.

### 2.4. Immunofluorescence Staining

The immunostaining of retinal cryosections was performed as described [25]. Slides were fixed in 4% paraformaldehyde and incubated overnight at 4 °C with anti-mouse primary antibodies used at the following concentrations: 4-hydroxynonenal (4-HNE) (1:100; Abcam, Cambridge, MA, USA), glial fibrillary acidic protein (GFAP) (1:200; Cayman Chemical, Ann Arbor, MI, USA), and RNA binding protein with multiple splicing (RBPMS) (1:500; GeneTex, Alton Pkwy Irvine, CA, USA). Slides were washed three times with 0.1% Triton X-100 in 0.1 M phosphate-buffered saline (PBS) (pH 7.4) followed by incubation with appropriate fluorescence-conjugated secondary antibodies (Life Technologies, Eugene, OR, USA). Sections were mounted using a fluoroshield mounting medium with DAPI (4′,6-diamidino-2-phenylindole; Sigma-Aldrich, St. Louis, MO, USA) and images captured at 20X magnification using Zeiss Axioplan-2 imaging fluorescence microscope (Carl Zeiss, Thornwood, NY, USA).

### 2.5. Immunoblotting

Total proteins were extracted from the retinas of mice using RIPA (Radioimmunoprecipitation assay) cell lysis buffer (Thermo Fisher, Waltham, MA, USA) containing 1% phosphatase and protease inhibitor cocktail (Sigma-Aldrich, St. Louis, MO, USA). An equivalent amount of protein samples (40–60 μg) were subjected to SDS–PAGE and transferred onto a PVDF (Polyvinylidene difluoride) membrane. Then, the membrane was blocked using 5% skimmed milk and incubated with the following anti-mouse primary antibodies: STAT3 phospho-tyrosine (705) (pSTAT3; 1:1000; Cell Signaling, Danvers, MA, USA), GFAP (1:1000; Santa Cruz Biotechnology, Dallas, TX, USA), zonula occludin-1 (ZO-1) (1:1000; Proteintech, Rosemont, IL, USA). After immunoblotting, the membranes were stripped using stripping buffer (Bio-Rad, Hercules, CA, USA) and re-probed with anti-β-actin antibody (1:3000; Sigma-Aldrich, St. Louis, MO, USA). Levels of pSTAT3 were normalized to total STAT3 (Signal transducer and activator of transcription 3). Chemiluminescence-based assay was used for band detection (Thermo Fisher, Waltham, MA, USA). Scanned images of blots were used to quantify protein expression using NIH ImageJ software (http://rsb.info.nih.gov/ij/). Assessment of VEGF_165_ protein levels was done using heparin affinity columns (Sigma-Aldrich, St. Louis, MO, USA) and Western blot analysis as previously described [20,23].

### 2.6. Dot Blot Snalysis

An equivalent amount of proteins prepared from whole mouse retinal lysates were spotted on nitrocellulose membranes and dried for 5 min at room temperature. The membranes were blocked for 1 h by using 5% skimmed milk in PBS and then probed for 1 h with either anti-3-nitrotyrosine (3-NT; 1:1000, Cayman, Ann Arbor, MI, USA) or anti-4-HNE (1:1000, Abcam, Cambridge, MA, USA) antibodies in PBS-tween buffer. Then, the membranes were washed three times in PBS-tween buffer and probed again with horseradish peroxidase-conjugate secondary antibody (1:5,000; Cell Signaling, Denvers, MA, USA). After washing the membrane, the immunopositive spots were visualized by using Clarity ECL Blotting substrate (Bio-Rad, Hercules, CA, USA).

### 2.7. Measurement of Retinal Vascular Leakage

To assess retinal vascular barrier function, Evans blue dye (EB) (Sigma-Aldrich, St. Louis, MO, USA) was used as described previously [26]. Mice were deeply anesthetized and EB dye, dissolved in normal saline (30 mg/mL), was injected intraperitoneally. The dye was allowed to circulate through the body for 2 h. For morphological studies, the eyes were enucleated and fixed in 4% paraformaldehyde at room temperature for 1 h, and then the retinas were excised, flat-mounted, examined, and imaged with a Zeiss Axioplan 2 fluorescence microscope (Carl Zeiss, Thornwood, NY, USA).

To quantify EB vascular leakage, harvested retinas were homogenized and incubated in formamide solution for 24 h at 55 °C (to extract EB). Following centrifugation, supernatants containing extracted EB were transferred into a 96-well plate, and absorbance was measured at 620 nm. The EB concentrations were calculated from a standard curve prepared using EB solution and presented as fold difference [27].

### 2.8. Dihydroethidium Staining for Detection of Superoxide

The dihydroethedium (DHE) method was used to evaluate superoxide formation as described previously [28]. Superoxide oxidizes DHE to ethidium bromide, which binds to DNA and fluoresces red. Briefly, retinal cryosections were brought to room temperature and subsequently covered with a 2 μM DHE solution. Slides were incubated in a light-protected humidified incubator at 37 °C for 30 min. At the end of the incubation, sections were mounted with a coverslip and images were captured immediately at 20X magnification using a Zeiss Axioplan-2 imaging fluorescence microscope (Carl Zeiss, Thornwood, NY, USA).

### 2.9. Cytokines Assay

To determine the levels of inflammatory cytokines in retinal lysate, we used a customized mouse Mix and Match Cytokine ELISA strip assay (Signosis, Santa Clara, CA, USA). Retinal tissue samples from different experimental groups were homogenized using cell lysis buffer (Signosis, Santa Clara, CA, USA), and total protein concentration was quantified using the Coomassie Plus (Bradford) Assay Kit (Thermo Fisher Scientific, Waltham, MA, USA). Samples containing an equivalent amount of proteins were added to different wells individually coated with primary antibodies against VEGF, TNFα (tumor necrosis factor α), and IL-6 (interleukin 6). Plates were processed according to the manufacturer’s instructions and were read at 450 nm. Protein standards provided by the manufacturer were used to calculate each cytokine concentration and expressed as ng/mg of total protein content.

### 2.10. Cells and Angiogenesis Assay

Human retinal microvascular endothelial cells (HuREC) were purchased from Cell System Corporation (Kirkland, WA, USA) and cultured according to the manufacturer’s instructions. All cells used for the experiments were between passages 3 and 5. In vitro angiogenesis assay was performed as described in [22]. HuREC cells were treated with 20 ng/mL VEGF alone or with different concentrations of UDCA for 6 h. At the end of treatment, the cells were trypsinized, and 300 μL of the cell suspension containing 1.0 × 10⁵ cells/well were added to 24-well plates, which were coated with growth factor reduced matrigel matrix (Corning Life Sciences, Tewksbury, MA, USA) following the manufacturer’s protocol. The angiogenesis assay plate was incubated at 37 °C, 5% CO₂ for 12 h. Captured digital images were analyzed for the extent of network formation by quantification of the number of interconnecting branching points. 

### 2.11. In Vitro Fluorescein Isothiocyanate–Dextran (FITC-Dextran) Leakage Assay

HuREC were seeded on a coated transwell membrane with 0.4 μm pores (Corning Costar, Glendale, AZ, USA), in complete growth media. Once attached, cells were treated with 20 ng/mL VEGF alone or in combination with 50, 100, or 200-μM UDCA for 24 h. The permeability of the cells in each treatment group was determined by measuring the apical-to-basolateral movement of 40 kDa FITC (Fluorescein isothiocyanate)-dextran (Sigma-Aldrich, St. Louis, MO, USA). The amount of FITC-dextran in the basolateral compartment was measured using a microplate reader (ELx800; Bio-Tek) set to 485 nm of excitation and 528 nm of emission [29] and expressed as fold change versus untreated cells.

### 2.12. Statistical Analysis

The results are presented as mean ± S.D for 3–6 replicates. Statistical significance of differences measured in the different treatment groups was evaluated using one-way analysis of variance (ANOVA), followed by Bonferroni’s multiple-comparison test, and results were considered significant when *p* < 0.05. The graphs were prepared using Graph Pad Prism version 8 software (GraphPad, San Diego, CA, USA).

## 3. Results

### 3.1. Differential Effects of Bile Acids on OIR in Mice

**Figure 1:**
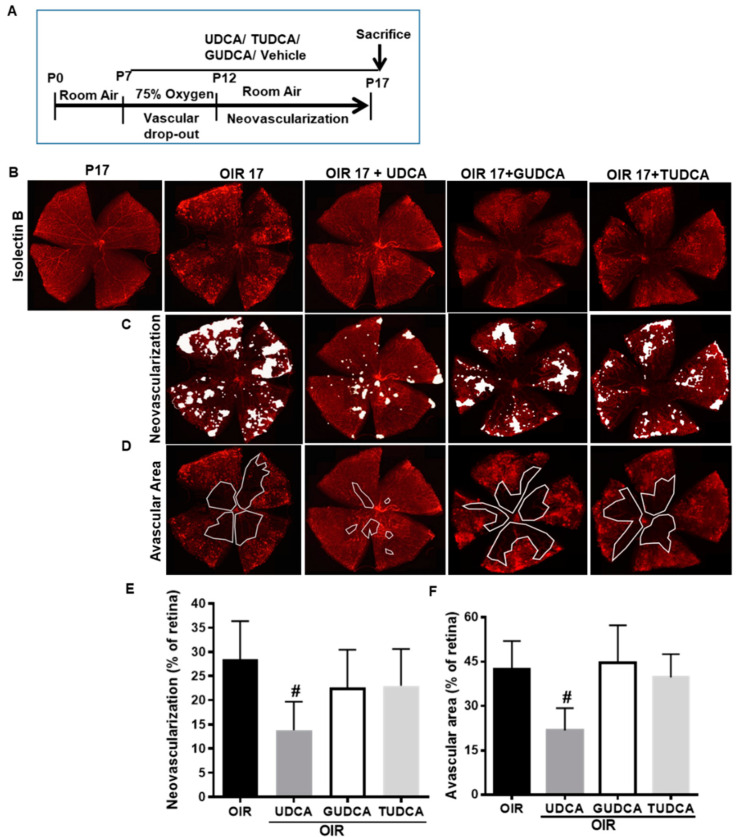
Effects of UDCA, TUDCA, and GUDCA treatment from P7 to P17 on pathological retinal neovascularization. (**A**) Overview of mouse OIR experimental model and different bile acids (BA) treatment paradigms. UDCA, TUDCA, and GUDCA (50 mg/kg) were administered by daily intraperitoneal injection from postnatal (P) days 7 to 17. All mice were euthanized at P17 and retinal flat mounts were stained with isolectin B4. (**B**) Representative retinal flat mounts from P17, OIR 17, and OIR17 treated with different BA (OIR 17 + UDCA, OIR 17 + TUDCA, and OIR17 + GUDCA). (**C**) The neovascular areas are indicated with white spots and the (**D**) area of avascular zones is highlighted with a white continuous line. (**E**,**F**) Histograms representing the results of morphometric analysis of retinal flat mounts measuring (**E**) area of neovascular tufts and (**F**) avascular areas. Values are mean ± S.D. (*n* = 6 retinas per group). # *p* < 0.05 vs. OIR. UDCA: ursodeoxycholic acid; TUDCA: tauroursodeoxycholic acid; GUDCA: glycoursodeoxycholic acid; OIR: oxygen-induced retinopathy.

UDCA, TUDCA, and GUDCA are the most widely used secondary conjugated BA for pharmacological treatments; hence, we evaluated the effects of these BA on retinal neovascularization in a mouse model of OIR. An outline of the OIR mouse model and treatment protocol is presented as Figure 1A. As shown in Figure 1B, isolectin B4 staining of retinal flat mounts showed that UDCA treatment (50 mg/kg), prolonged for all OIR time (P7–P17), resulted in a significant reduction of retinal neovascularization (Figure 1C) and amelioration of vascular distribution (Figure 1D). In particular, morphometric analysis showed that UDCA decreased over 50% of the neovascular area (*p* < 0.011 versus OIR) (Figure 1E) and the extension of the avascular area (*p* < 0.002 versus OIR) (Figure 1F). On the contrary, treatments of OIR mice with GUDCA and TUDCA that were used at the same doses and for the same treatment times failed to show any significant change in neovascular area (Figure 1E) or avascular areas (Figure 1F) when compared to untreated OIR mice.

### 3.2. Effect of UDCA Treatment during Hyperoxic and Hypoxic Phase on OIR in Mice

**Figure 2:**
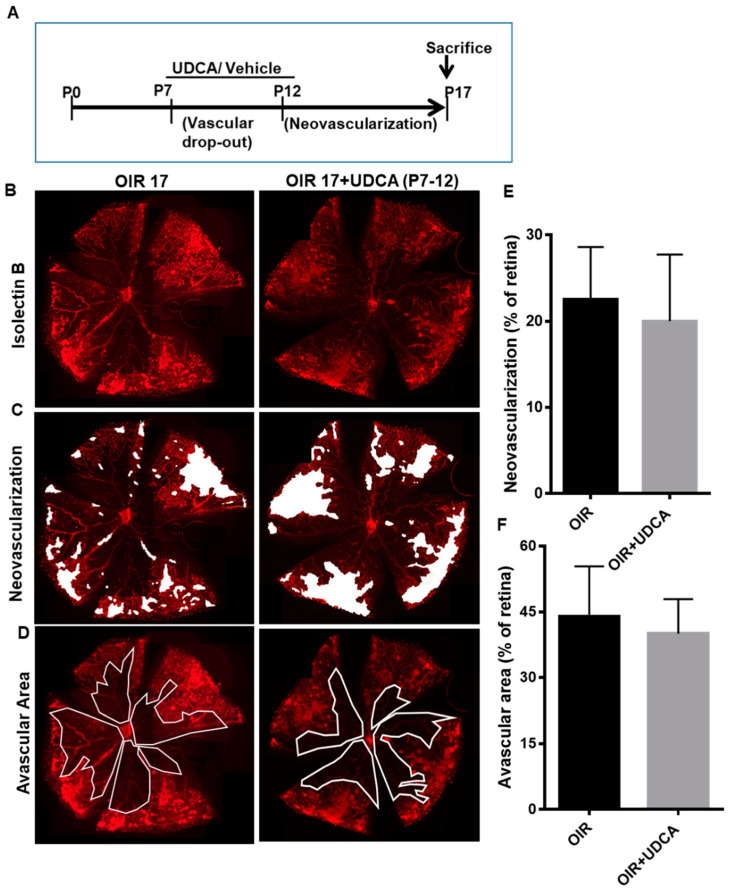
Effect of UDCA treatment during the hyperoxic phase on pathological neovascularization in OIR mice. (**A**) Schematic diagram depicting mouse OIR experimental model and UDCA (50 mg/kg) treatment from P7 to P12. (**B**) Representative flat-mounted retinas of OIR mice at P17 and OIR mice treated with 50 mg/kg UDCA, from P7 to P12, stained with isolectin B4 to identify (**C**) areas of neovascularization (white spots) and (**D**) avascular area (white lines). Histograms representing the results of morphometric analysis of retinal flat mounts measuring (**E**) area of neovascularization and (**F**) avascular area. Values are mean ± S.D. (*n* = 6 retinas per group).

**Figure 3:**
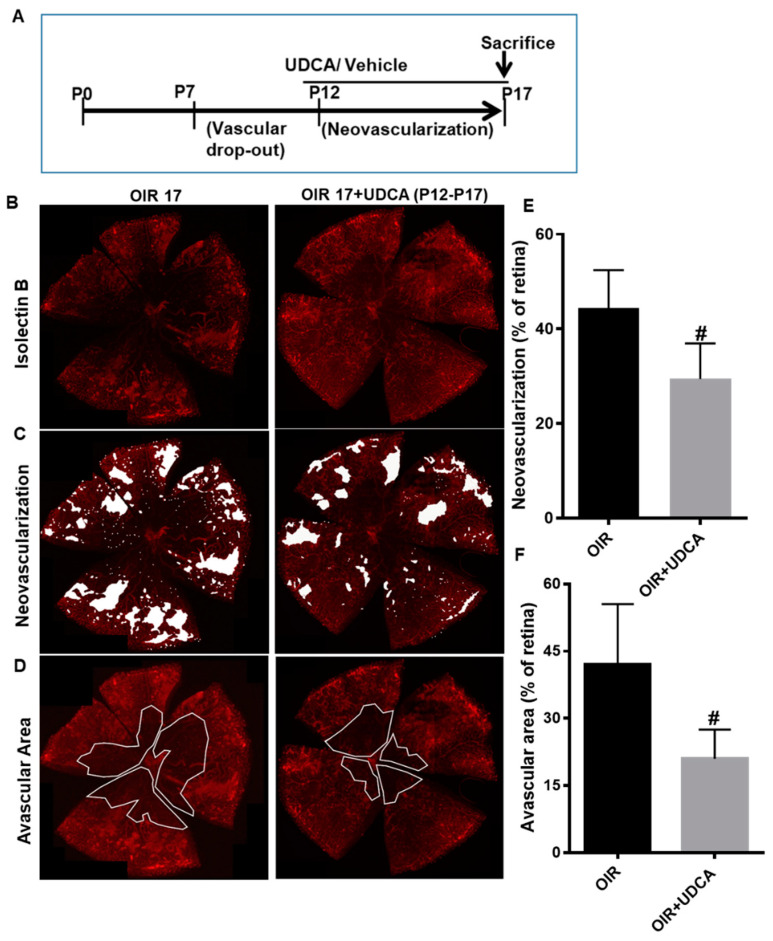
Effect of UDCA treatment during the hypoxic phase on pathological neovascularization in OIR mice. (**A**) Schematic diagram depicting mouse OIR experimental model and UDCA (50 mg/kg) treatment from P12 to P17. (**B**) Representative images of isolectin-B4 stained flat-mounted retinas of OIR mice at P17 and of OIR mice treated with 50 mg/kg UDCA from P12 to P17 to identify areas of neovascularization (white spots) (**C**) and avascular zones (white lines) (**D**) Histograms representing the results of morphometric analysis of retinal flat mounts measuring (**E**) area of neovascularization and (**F**) avascular area. Values are mean ± S.D. (*n* = 6 retinas per group). # *p* <0.05 vs. OIR.

To better understand UDCA effects, we next examined the results of UDCA administration during either the hyperoxic or the hypoxic phase of the OIR mouse model. As outlined in Figure 2A, mice were treated with 50 mg/kg UDCA from P7 to P12 (hyperoxia phase) or kept untreated. All the treatment groups were exposed to 75% oxygen from P7 to P12 and room air from P12 to P17. Evaluation of isolectin B4 staining of retinal flat mounts, performed on OIR mice at P17, showed no significant morphological differences between untreated and UDCA-treated OIR mice (Figure 2B–D). Quantification of retinal neovascularization and/or avascular areas did not show differences (Figure 2E,F). In another set of experiments, mice were treated with 50 mg/kg UDCA from P12 to P17 (hypoxia phase) or kept untreated (Figure 3A). All the treatment groups were exposed to 75% oxygen from P7 to P12 and room air from P12 to P17. In this case, UDCA treatment imposed during the hypoxic phase (OIR12–OIR17) normalized retinal morphology (Figure 3B–D), as also confirmed by significant reductions of both neovascular tufts (*p* < 0.01 versus OIR) and avascular area (*p* < 0.01 versus OIR) (Figure 3E,F). 

### 3.3. UDCA Preserves Blood–Retinal Barrier (BRB) in OIR Mice

**Figure 4:**
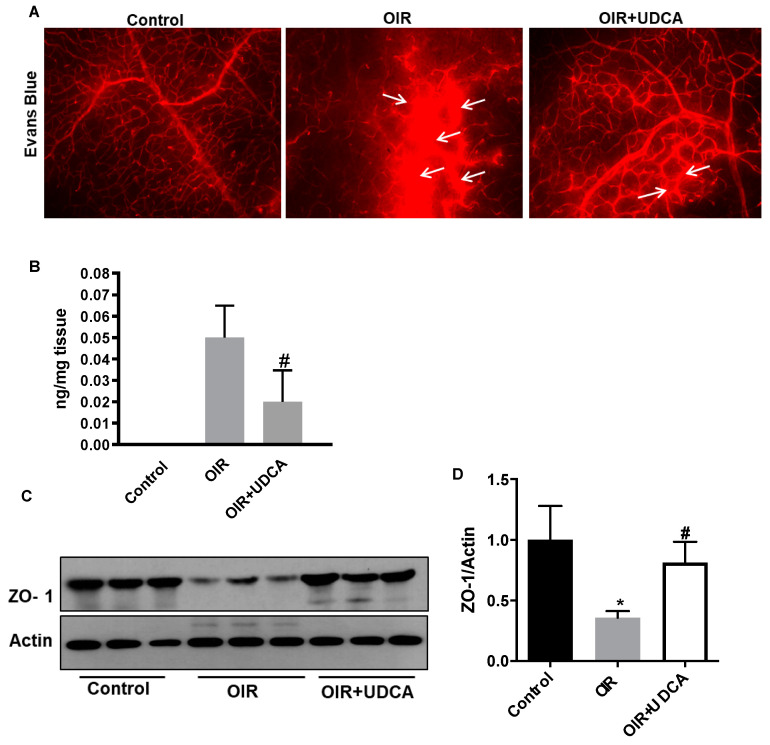
UDCA preserves blood–retinal barrier in OIR mice. (**A**) Evans blue (EB) dye extravasation (white arrows) assessed at P17, in control mice (room air), OIR mice, and OIR+UDCA mice (treated with 50 mg/kg UDCA from P7-P17). Two hours after EB injection, eyes were collected to prepare flat mounts and examined using a fluorescence microscope. (**B**) In a separate set of experiments, EB-injected mice were perfused with phosphate-buffered saline (PBS), and the residual dye in the retina was quantified following formamide extraction. (**C**) Representative image of zonula occludin-1 (ZO-1) immunoblotting from the different treatment groups. (**D**) Densitometry results showing changes in ZO-1 protein expression normalized to the loading control actin. Values are mean ± S.D. (*n* = 6 retinas per group). * *p* < 0.05 vs. control and # *p* < 0.05 vs. OIR.

Based on the obtained results suggesting an anti-angiogenic effect of UDCA on the postnatal retinal vasculature, we have further continued our investigation assessing the impact of UDCA treatment on other important OIR pathological features associated with the occurrence of ROP. First of all, we have assessed the effects of UDCA on vascular permeability, as measure of BRB integrity, in OIR mice using Evans blue (EB) leakage assay. EB dye (30 mg/mL) was injected i.p., and 2 h after, the eyes were enucleated and retinal flat mounts were examined to visualize dye extravasation. After i.p. injection, EB rapidly binds to plasma albumin and in case of compromised vascular permeability, the EB dye–albumin complex leaks into the surrounding tissues. In retinal flat mounts, we observed elevated EB extravasation in OIR mice compared to control mice (Figure 4A, white arrows). However, UDCA treatment significantly down-regulated OIR-induced EB extravasation, thus indicating a prevention of BRB breakdown (Figure 4A). The quantification of EB extravasation following formamide extraction of perfused mice retinas confirmed the imaging data showing that UDCA treatment significantly reduced EB residual retinal content (*p* < 0.006 versus OIR) (Figure 4B). Increased vascular permeability and a consequent breakdown of BRB results from altered expression and function of intercellular junction proteins such as zonula occludens-1 (ZO-1) [30]. Western blotting (Figure 4C) and densitometric analyses (Figure 4D) showed that ZO-1 protein levels were significantly reduced in OIR mice (*p* < 0.02 versus control) and that UDCA treatment significantly preserved ZO-1 levels (*p* < 0.02 versus OIR).

### 3.4. UDCA Treatment Diminishes Reactive Gliosis and Rescues Neuronal Cells in OIR Mice

**Figure 5:**
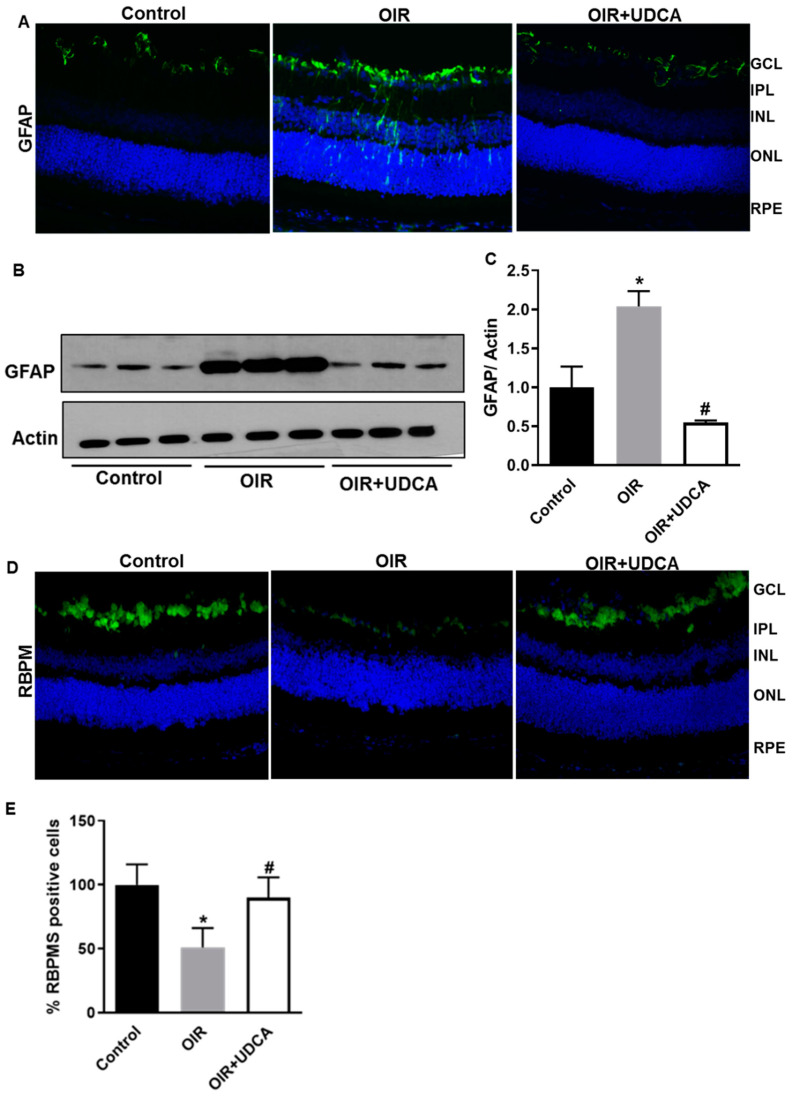
UDCA treatment halts reactive gliosis and preserves neuronal cells in OIR mice. Frozen retinal sections from control mice at P17 (room air), OIR mice, and OIR mice treated with 50 mg/kg UDCA (P7–P17) were stained with (**A**) GFAP antibody. Total protein fractions from control mice (room air), OIR mice at P17, and OIR mice treated with 50 mg/kg UDCA (P7–P17) were used to analyze (**B**) protein levels of GFAP by Western blotting. (**C**) Densitometry graphs showing changes in glial fibrillary acidic protein (GFAP) protein expressions. Values are mean ± S.D. (*n* = 6 retinas per group). (**D**,**E**) Frozen retinal sections from control mice at P17 (room air), OIR mice, and OIR mice treated with 50 mg/kg UDCA (P7–P17) were stained with RNA binding protein with multiple splicing (RBPMS) antibody and positive cells were counted. RBPMS data are expressed as cell number/100 μm retinal length and presented as a percent change from control. * *p* < 0.05 vs. control and # *p* < 0.05 vs. OIR. GLC: ganglion cell layer; IPL: inner plexiform layer; INL: inner nuclear layer; ONL: outer nuclear layer; RPE: retinal pigmented epithelium; RBPMS: RNA Binding Protein with Multiple Splicing; GFAP: Glial fibrillary acidic protein.

Reactive gliosis and loss of retinal ganglion cells are features associated with OIR in mice [31]. In agreement with previous reports, we observed significant reactive gliosis in OIR mice, as indicated by increased GFAP immunoreactivity stretching through the entire retina (Figure 5A). Western blotting (Figure 5B) and densitometric analyses (Figure 5C) confirmed the immunohistochemical data. UDCA treatment significantly (*p* < 0.0002) reduced gliosis as indicated by lower GFAP levels in UDCA-treated mice compared to OIR (Figure 5A–C). Further, to assess the effects of UDCA on retinal neurons in OIR mice, we performed immunohistochemical analysis using the specific ganglion cell marker RNA binding protein with multiple splicing (RBPMS) [32]. The results of this analysis (Figure 5D,E) showed a marked decrease in RBPMS-specific immunoreactivity in OIR mice retinas (Figure 5D) that was restored by UDCA treatment (Figure 5D). These data were confirmed by counting RBPMS positive cells throughout the retinal ganglion cell layer and expressed as percentage of control (Figure 5E).

### 3.5. Effect of UDCA Treatment on Oxidative Stress in OIR Mice

**Figure 6:**
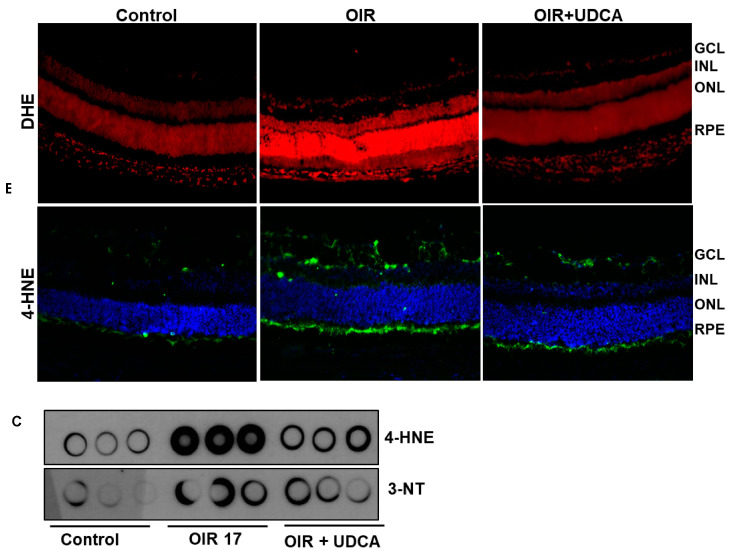
Effect of UDCA treatment on oxidative stress in OIR mice. Frozen retinal sections from control mice at P17 (room air), OIR mice, and OIR mice treated with 50 mg/kg UDCA (P7–P17) were stained with (**A**) dihydroethedium (DHE) and (**B**) 4-hydroxynonenal (4-HNE) to assess oxidative stress. (**C**) Dot blot assay assessing 4-HNE and 3-nitrotyrosine (3-NT) levels from total protein fractions extracted from control mice (room air), OIR mice at P17, and OIR mice treated with 50 mg/kg UDCA (P7–P17).

Oxidative stress is a critical pathogenic event for the development of OIR and RNV. Therefore, we sought to evaluate the effects of UDCA on retinal oxidative stress in OIR mice. The detection of superoxide formation with DHE staining showed that UDCA treatment halted superoxide increase in OIR mice (Figure 6A). In addition, 4-HNE immunostaining of retinal sections showed decreased immunoreactivity of this lipid peroxide in UDCA-treated mice (Figure 6B). Finally, dot blot analysis of retinal extracts confirmed the effects of UDCA in decreasing oxidative/nitrative stress by showing a decreased immunoreactivity of 4-HNE and 3-nitrotyrosine (3-NT) in OIR mice treated with UDCA (Figure 6C).

### 3.6. UDCA Regulates STAT3 Signaling to Reduce Inflammation in OIR Mice

**Figure 7:**
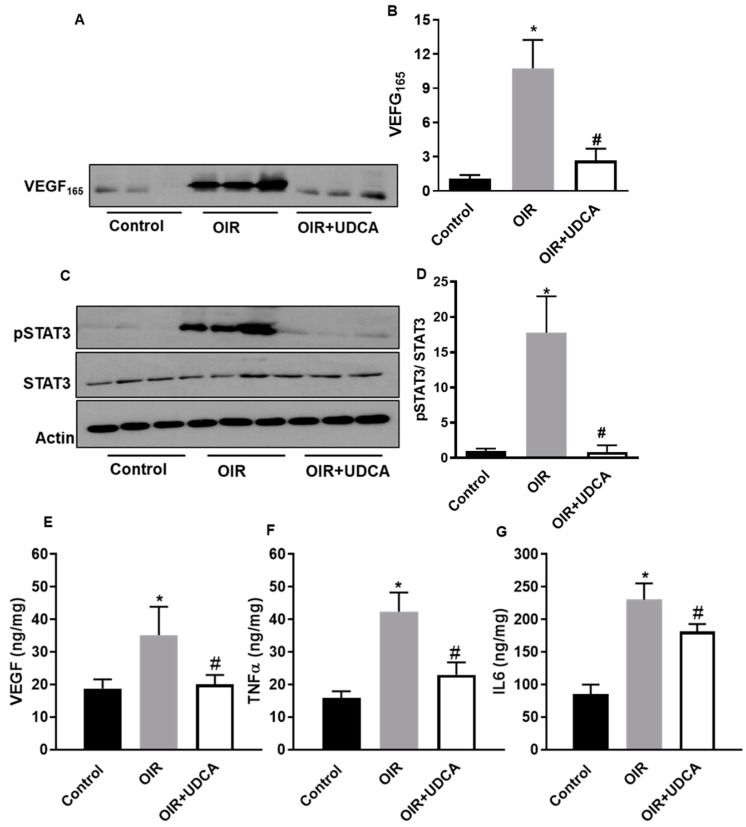
UDCA regulates STAT3 (Signal transducer and activator of transcription 3) signaling to reduce inflammation in OIR mice. Total protein fractions from control mice (room air), OIR mice, and OIR mice treated with 50 mg/kg UDCA (P7–P17) at P17 were used to analyze protein levels of VEGF₁₆₅ (**A**), pSTAT3, and STAT3 (**C**) by Western blotting. (**B**–**D**) Densitometry graphs showing changes in protein expressions. (**E**–**G**) The total retinal extracts were used to quantify protein levels of vascular endothelial growth factor (VEGF), tumor necrosis factor α (TNFα), and interleukin 6 (IL-6) using ELISA assay. Values are mean ± S.D (*n* = 6 retinas per group). * *p* < 0.05 vs. control and # *p* < 0.05 vs. OIR.

Increased VEGF expression is a main contributing factor in inducing and sustaining RNV. Further, VEGF-induced STAT3 activation has been implicated in exacerbated inflammation in OIR mice retinas [20,21,23]. Hence, we evaluated the effect of UDCA treatment on VEGF-induced STAT3 signaling and inflammation in OIR mice. As previously reported, OIR mice had significantly elevated levels of VEGF (*p* < 0.002 versus control) [20,33,34], and UDCA treatment was able to reduce this increment (*p* < 0.005 vs OIR) (Figure 7A,B and E). Further, VEGF-induced phosphorylation of STAT3 was significantly reduced in retinas of UDCA-treated mice (*p* < 0.01 vs OIR) (Figure 7C,D). In agreement with changes in VEGF levels and STAT3 phosphorylation, retinas of ROP mice had significantly higher levels of inflammatory cytokines such as TNFα (Tumor necrosis factor alpha) and IL-6 (Interleukin 6; *p* < 0.001 versus control). However, UDCA treatment significantly reduced the levels of these inflammatory mediators (*p* < 0.001 versus OIR) (Figure 7F,G).

### 3.7. Effect of UDCA Treatment on VEGF-induced Pro-Angiogenic and Hyperpermeability Effects

**Figure 8:**
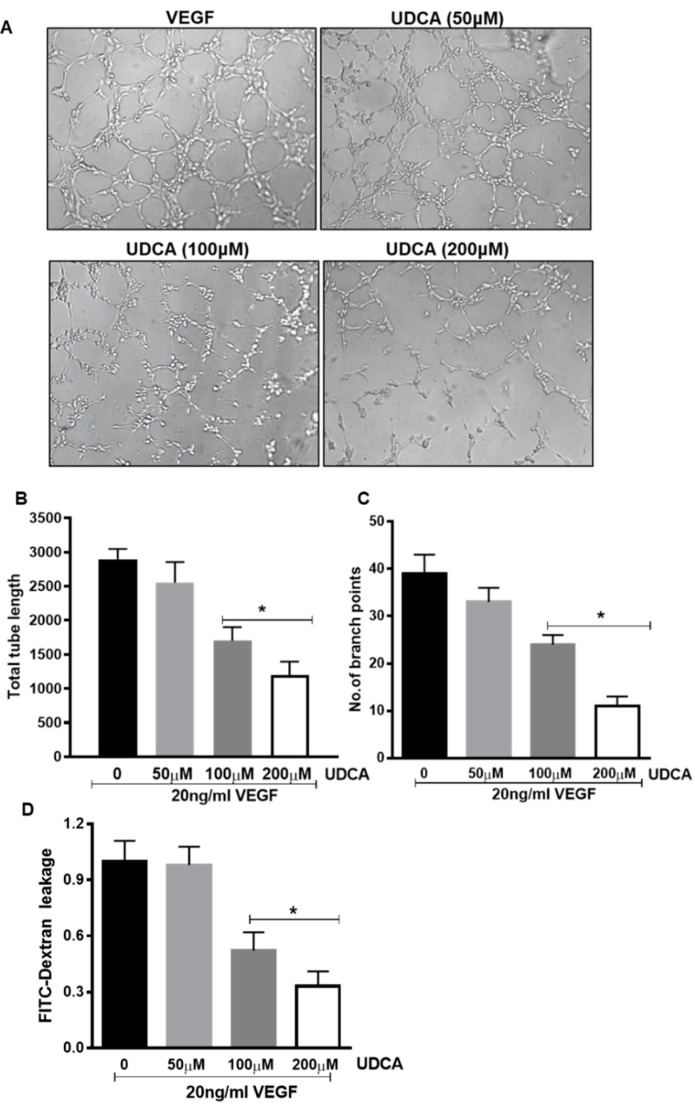
Effect of UDCA on in vitro tube formation and barrier function in VEGF-treated human retinal microvascular endothelial cells (HuREC). HuREC were maintained on matrigel-coated plates in the presence of 20 ng/mL VEGF alone or in combination with UDCA (50–200 μM). (**A**) Micro images of tube formation assay. (**B**,**C**) Graphs representing results of (**B**) tube length and (**C**) number of branch points. Values are mean ± S.D (*n* = 3). (**D**) Measurements of FITC (fluorescein isothiocyanate) fluorescence from HuREC grown in trans-well plates after treatment with 20 ng/mL VEGF in presence of different UDCA doses (50–200 μM). * *p* < 0.05 vs. cells not treated with UDCA.

Based on UDCA effects on the hypoxic/neovascularization phase and on the VEGF–STAT3 axis observed in the OIR mice, we have assessed the potential impact of UDCA directly on VEGF-dependent pro-angiogenic and permeability function in HuREC in culture. In vitro tube formation assay was conducted to determine the effects of various concentrations of UDCA on VEGF-induced angiogenic response in HuREC. To account for off-side effects, we have monitored all UDCA in vitro tested doses using MTT (3-(4,5-dimethylthiazol-2-yl)-2,5-diphenyl tetrazolium bromide) assay and the obtained results, shown in Figure S1, confirmed that these were not intrinsically toxic to HuREC. As shown in Figure 8A, the treatment of HuREC with 20 ng/mL VEGF for 6 h significantly induced the formation of tube-like structures. Co-treatment with different doses of UDCA (50–100–200μM) progressively halted VEGF-induced tube formation in HuREC with significant changes observed in response to 100 and 200 μM (Figure 8A). Calculation of total number of branch points and length of the tubes further revealed a 40–60% reduction (*p* < 0.001 versus VEGF) of VEGF effects by UDCA treatment (Figure 8B,C). Furthermore, we assessed UDCA effects on VEGF-induced FITC-dextran leakage in HuREC monolayers cultures on trans-wells. Treatment of HuREC cells with 20 ng/mL of VEGF significantly compromised barrier resulting in a leakage of FITC-dextran in the lower compartment of a trans-well plate. While, 50 μM UDCA had no effect, 100 and 200 μM UDCA significantly (*p* < 0.01 and *p* < 0.001, respectively, versus VEGF) reduced VEGF-induced FITC-dextran leakage across the membrane (Figure 8D).

## 4. Discussion

ROP, the leading cause of blindness in children, is an ocular manifestation in premature babies with limited and potentially invasive therapeutic options [10,35]. Halting ROP in premature children is critical for the prevention of severe ocular complications of the affected children that could eventually result in severe hypovision or complete blindness [36,37,38]. Herein, we have investigated the effects of the secondary BA, UDCA, and its glycine and taurine derivatives, GUDCA and TUDCA, in halting ROP-like features in mice subjected to OIR. Besides their well-known role as regulators of hepatic cholesterol homeostasis, these important bioactive molecules have recently gained attention for the treatment of a number of ocular diseases—in particular, retinal ischemic pathologies such as diabetic retinopathy [39,40]. To date, little is known on the effects of these highly biological active compounds for the treatment of pediatric eye diseases such as ROP. The results of our studies show that UDCA treatment of OIR mice from P7 to P17 resulted in amelioration of the retinal vascular phenotype, as shown by decreased neovascular tufts areas in OIR-treated mice. Most importantly, UDCA diminished the extension of the avascular area, thus demonstrating anti-angiogenic effects without blocking normal revascularization of the central retina. These effects were confirmed when UDCA was administered only during the hypoxic phase (P12–P17), but not the hyperoxic stage (P7–P12), thus revealing the anti-angiogenic properties of this compound on the ischemic retinal vasculature. Concomitant with these findings, we also found that UDCA prevented reactive gliosis, loss of neuronal cells, and breakdown of the BRB, which are all key features of OIR and ROP [31,41,42,43]. To confirm the anti-angiogenic ability of UDCA, we also determined that this BA decreases VEGF expression in the OIR retinas and prevents VEGF pro-angiogenic and pro-permeability effects in HuREC in vitro. Hypoxia-induced VEGF secretion promotes angiogenesis, vascular permeability, and inflammation, which are all features that are involved in the progression of ischemic retinopathy [43]. UDCA treatment decreased the expression of VEGF and of inflammatory cytokines (TNFα and IL-6) concomitantly with the inhibition of STAT3 [20,23]. This transcription factor is a key mediator of cellular responses to inflammatory stimuli and a downstream effector of VEGF activity in endothelial cells [21,37]. Our group and others have previously shown that VEGF expression and activity is tightly regulated by STAT3 and that its inhibition halts VEGF-mediated pro-angiogenic effects [21,34]. Of interest, STAT3 activation in the ischemic retina and in retinal endothelial cells has been shown to result from increased oxidative/nitrative stress [33,44]. In the present studies, UDCA treatment resulted in a significant decrease of the oxidative/nitrative stress markers 4-HNE and 3-NT, and this effect could result in the blockade of phosphorylation/activation of STAT3 and STAT3-dependent VEGF expression and signaling [20,33]. Due to the key role of VEGF in promoting RNV in the ischemic retina [45], UDCA’s anti-angiogenic effects in OIR mice could be attributable, at least in part, to its effects in halting the VEGF–STAT3 axis by attenuating oxidative/nitrative stress signals in the ischemic retina. Similarly, UDCA has been shown to improve other ocular pathologies that involve excessive VEGF action (or oxidative stress) such as wet age-related macular degeneration [46] and diabetic retinopathy [40], thus, further highlighting the significant impact of UDCA on VEGF signaling.

Finally, of particular interest is our finding that despite the strict structural similarities, treatments with same doses of GUDCA and TUDCA (P7–P17) had no effects in OIR mice, thus suggesting that the postnatal retinal vasculature displays selective sensitivity to different BA. Remarkably, these compounds bind different receptors [47]. UDCA, which can bind FXR (Farnesoid X receptor) and TGR5 [48,49,50], prevented retinal neovascularization in OIR mice, whereas TUDCA and GUDCA that bind only TGR5 (G-protein-coupled bile acid receptor, Gpbar1) [51,52] had no effects, thus warranting further studies to analyze the expression pattern and biological activities of different BA receptors in the postnatal retina and developing retinal microvasculature.

## 5. Conclusions

The therapeutic treatment of ROP is particularly challenged by the use of invasive anti-RNV therapies often resulting in the blockade of normal postnatal retinal vasculogenesis and damage to the neuroretina [53]. The results of our studies demonstrate that UDCA can halt pathological neovascularization in the ischemic postnatal retina without affecting normal vascular growth or provoking systemic toxicity (Figure S2), thus satisfying two very important characteristics of a drug candidate for the treatment of ROP. Importantly, UDCA-containing drug formulations are already available and their use could be rapidly repurposed for ROP applications.

## Supplementary Materials

The following are available online at https://www.mdpi.com/2077-0383/9/6/1921/s1, Figure S1: Effect of UDCA on the viability of Human retinal endothelial cells, Figure S2: UDCA does not induce systemic toxicity in mice.
