# Supplementary material for: Ursodeoxycholic Acid Halts Pathological Neovascularization in a Mouse Model of Oxygen-Induced Retinopathy
Source: J Clin Med. 2020 Jun 19;9(6):1921. doi: 10.3390/jcm9061921 (PMC7356323; doi:10.3390/jcm9061921)
Supplement: Supplementary file 1 [file jcm-09-01921-s001.pdf]

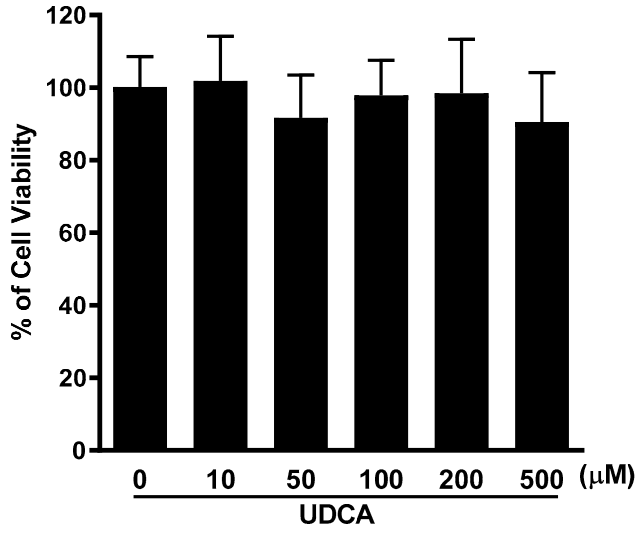

Figure. S1 Effect of UDCA on the viability of Human retinal endothelial cells (HuREC).
HuREC cells were exposed to different concentrations of UDCA (10-500μM) for 24 hours and cell viability was evaluated using MTT assay. Values are mean ± S.D (n=6 retinas per group)

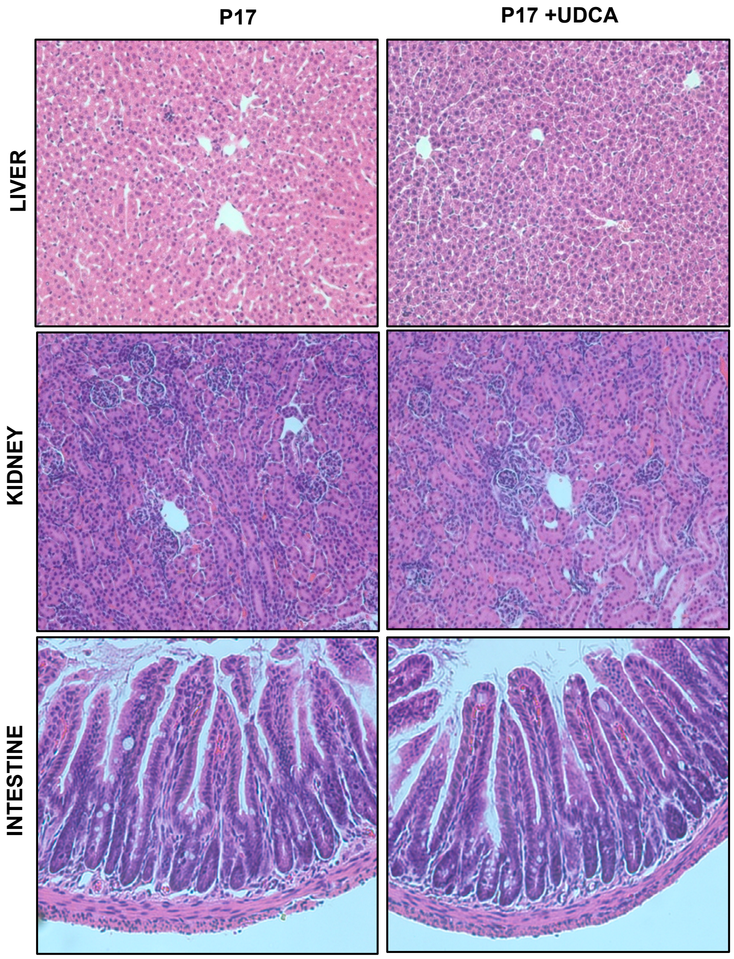

Figure. S2 UDCA does not induce systemic toxicity in mice.

Mice were treated with either vehicle (PBS) or 50 mg/kg (i.p. daily) UDCA from postnatal day 7 to 17. At the end of the treatment, liver kidney and intestine were collected, fixed in paraformaldehyde and embedded in paraffin. Paraffin embedded tissues were sectioned and stained with hematoxylin and eosin.
